# A Narrative Review of RAS Mutations in Early-Stage Colorectal Cancer: Mechanisms and Clinical Implications

**DOI:** 10.3390/medicina61030408

**Published:** 2025-02-26

**Authors:** Hasan Cagri Yildirim, Damla Gunenc, Elvina Almuradova, Osman Sutcuoglu, Suayib Yalcin

**Affiliations:** 1 Department of Medical Oncology, Nigde Training and Research Hospital, 51100 Nigde, Turkey; 2 Department of Medical Oncology, Ege University Faculty of Medicine, 35040 Izmir, Turkey; damla.gunenc@ege.edu.tr; 3 Medicana Hospital, 34520 Istanbul, Turkey; elvina.almuradova@ege.edu.tr; 4 Department of Medical Oncology, Etlik City Hospital, 06170 Ankara, Turkey; sutcuogluo@gmail.com; 5 Department of Medical Oncology, Hacettepe University Faculty of Medicine, 06230 Ankara, Turkey; suayibyalcin@yahoo.com

**Keywords:** colon cancer, KRAS mutation, MSI/MSS status, prognostic markers, early-stage, survival

## Abstract

Colorectal cancer (CRC) is the third-most common cancer globally and a leading cause of cancer-related deaths. While the prognostic and predictive roles of RAS mutations in advanced CRC are well-established, their significance in early-stage CRC remains a topic of debate. Studies have been conducted for many years on clinical and pathological parameters that may be associated with RAS mutation, and there are inconsistent results in this regard. Currently, the only biomarker used in early-stage CRC is microsatellite status. KRAS mutations are detected in 40–50% of patients with colorectal cancer. RAS activating mutations cause loss of EGFR regulation by acting on the RAS/RAF/MAPK signaling pathways. In advanced colorectal cancer, these mechanisms cause a decrease in the effectiveness of EGFR inhibitors. However, studies on patients with early-stage colorectal cancer have inconsistent results. This review highlights the prognostic and clinical significance of KRAS mutations in early-stage CRC, particularly in MSS tumors. In the MSS group, KRAS mutations were associated with shorter TTR and OS compared to DWT patients. In contrast, in the MSI-H group, KRAS mutations showed no prognostic effect in TTR and OS. However. KRAS mutations were associated with shorter SAR in both MSI-H and MSS groups of patients. The findings underscore the need for routine molecular profiling, including KRAS and MSI status, to refine risk stratification and guide adjuvant therapy decisions. Further studies are warranted to explore targeted therapeutic approaches for KRAS-mutant CRC in the adjuvant setting.

## 1. Introduction

Colorectal cancer (CRC) is the third-most common cancer in the world and ranks only second after lung cancer in cancer-related deaths [1]. According to GLOBOCAN data in 2022, more than 1.9 million individuals were diagnosed with CRC, accounting for approximately 10% of all new cases of cancer worldwide [2]. In 2040, it is estimated that there will be 3.2 million new cases and 1.6 million colorectal cancer-related deaths worldwide [3]. CRC is a highly heterogeneous malignancy, and many well-known genetic alterations and signaling pathway pathologies are involved in its mechanism [4]. While 75% of CRCs are sporadic, genetic or familial factors play a role in 25–30% [5,6]. Somatic or genetic mutations in the adenomatous polyposis coli (APC) gene are frequently detected in early carcinogenesis in the development of CRC and are detected in 70% of patients with adenoma [7]. This mutation is often followed by activating mutations in the Kirsten rat sarcoma viral oncogene homolog (KRAS) and inactivating mutations in the TP53 and SMAD4 tumor suppressor genes driving the progression to carcinoma [8]. SMAD4-mediated bone morphogenetic protein (BMP) signaling inhibits intestinal tumorigenesis, whereas SMAD4-independent BMP signaling promotes metastasis in colorectal tumors [9,10]. p53 encodes a protein that regulates the cell cycle, DNA repair, aging, and apoptosis [11].

The RAS gene family is widely expressed in mammalian cells, encoding four small (21 kDa) cytoplasmic proteins with GTPase activity: Harvey rat sarcoma viral oncogene homolog (HRAS), K-RAS4a, K-RAS4b, and Neuroblastoma RAS viral oncogene homolog (NRAS) [12]. RAS mutations are a significant focus in oncology and have historically been associated with resistance to anti-epidermal-growth-factor-receptor (EGFR) therapies. Although the prognostic and predictive impact of RAS mutations in advanced CRC are well-established, their significance in early-stage CRC remains a topic of debate [13]. Herein, we conducted a literature review and critically discussed the significance of KRAS mutation in early-stage CRC in the context of recent advances and future directions.

## 2. Methods

We first discussed the RAS mutation mechanism, then the clinical factors that may be associated with RAS mutation, and finally the effect of RAS mutation on early-stage CRC prognosis. In our study, the keywords “early stage colorectal cancer”, “early stage colon cancer”, “early stage rectal cancer”, and “RAS mutation” were searched among the articles written in English using the PUBMED database.

## 3. Mechanism of RAS Mutation

**Figure 1:**
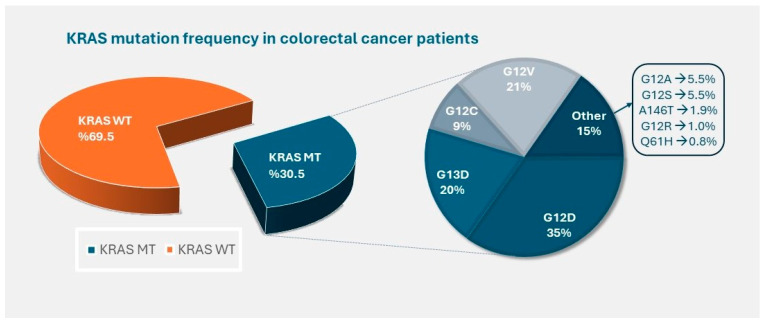
Data for KRAS point mutations was collected from the COSMIC somatic mutations database v100 using auto-filtering “large intestinal” for tissue type, “colon” for subsite, “carcinoma” for histology, and “adenocarcinoma” for sub-histology. MT, mutant; WT, wild-type.

Based on data from The Cancer Genome Atlas, the mutation rate in the Receptor tyrosine kinase-RAS network varies between 30% and 96% depending on the type of cancer [14]. Among these, KRAS is the most frequently mutated oncogene in cancers. Activating KRAS mutations are most prevalent in pancreatic cancer, occurring in approximately 90% of cases, followed by 40–50% of CRC patients and only about 7% of prostate cancer cases. KRAS mutation frequency in CRC is shown in Figure 1 [15,16]. NRAS mutations are detected in 1.2–4.2% of CRC. However, Harvey rat sarcoma viral oncogene homolog (HRAS) mutations are extremely rare in CRC, and unlike KRAS and NRAS, their clinical significance remains unclear [17,18]. The most common KRAS mutations are KRAS G12D (35%), followed by KRAS G12V (21%), KRAS G13D (20%), and KRAS G12C (9%).

**Figure 2:**
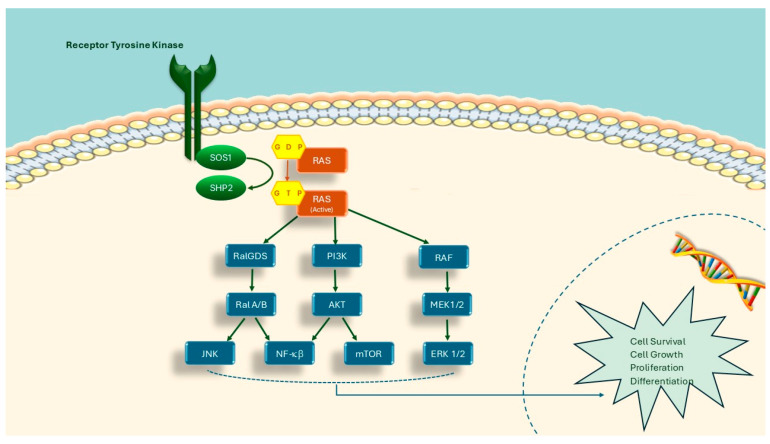
RAS signaling pathways.

KRAS protein acts as an endogenous nucleotidase, guanosine triphosphatase (GTPase), that interacts with receptor tyrosine kinases on the cell membrane, leading to cell proliferation and inhibition of apoptosis, switching between inactivated GDP-bound and active GTP-bound forms [19]. Most KRAS somatic mutations disrupt endogenous GTPase activity, causing an increase in RAS-GTP [19]. The increase in the active GTP-bound form plays a role in carcinogenesis by activating effector proteins such as RAF-kinases, phosphatidylinositol 3-kinase (PI3K), and RalGDS. KRAS activating mutations also, play an important role in the EGFR signaling pathway by activating the RAS/RAF/MAPK signaling pathways and causing loss of EGFR regulation (RAS signaling pathways are shown in Figure 2) [20]. Since RAS mutations are so common in the pathogenesis of many cancers and remain an area of active investigation regarding prognostic and predictive significance, as well as their potential as therapeutic targets, the RAS Initiative project, supported by the National Cancer Institute (NCI) in the USA, was launched in 2013. The main goal of this project is to unite the community and provide shared resources to create a greater focus on understanding RAS biology and foster innovation, advancing drug development through cutting-edge science. As part of this project, the production, purification, and determination of the crystal structure of fully processed KRAS4b, a splice variant of KRAS, provided critical insights. KRAS4b has been linked to larger tumor sizes and a worse prognosis compared to KRAS4a [21]. Other studies conducted by this project include: “Structural dynamics of RAF1-HSP90-CDC37 and HSP90 complexes”, “Revealing the mechanism of action of a first-in-class covalent inhibitor of KRASG12C (ON) and other functional properties of oncogenic KRAS by (31)P NMR”, “RAS and SHOC2 roles in RAF activation and therapeutic considerations” [22,23,24]. To date, this project team has made substantial contributions to the literature, publishing numerous articles, significantly enhancing our understanding of KRAS [25,26,27].

More than 3000 KRAS point mutations have been identified in CRC, with the majority located in exon 2 (codons 12 and 13), exon 3 (codon 61), or exon 4 (codon 146) [28,29]. Among these mutations, codon 12 is the most common location, accounting for 65% of cases, followed by codons 13, 61, and 146, with rates of 17%, 2%, and 4%, respectively [30]. KRAS codon 12 mutations are most frequently observed in G12D and G12V subtypes [31]. Submolecular analyses are important because subtypes have distinct clinical significance [32]. KRAS G12C mutation, characterized by the substitution of glycine to cysteine, is detected in approximately 2–4% of all CRC patients [33,34,35]. Studies comparing the prognosis of patients with KRAS G12C or G12V mutation with other KRAS (non-G12C/V) mutations have shown that KRAS G12C or G12V mutant patients may have poorer progression-free survival (PFS) and overall survival (OS) [36,37,38,39]. However, the prognostic significance of specific KRAS mutations remains an area of ongoing research, and conflicting results have been reported. Both, KRAS G12C and KRAS G12V bind to the RAL guanine nucleotide dissociation stimulator (RALGDS) (a RAL GTPase-specific guanine nucleotide exchange factor), suggesting a potential therapeutic target [40]. KRAS G12D is the most common KRAS mutation, detected in 10–12% of all CRC cases, and is associated with high expression of PI3K [40,41]. According to the study by Zlobec et al., KRAS G12D mutation had a poorer prognosis than other subgroups [42]. Guerrero et al. evaluated the prognostic effect of KRAS codon 12 and 13 mutations. According to the results of this study, codon 13 mutations have more aggressive clinical course than codon 12 [43]. In contrast, in a study published in 2010, the prognosis of KRAS G13D mutant patients receiving supportive care was similar to non-KRAS G13D mutant patients and also those with KRAS wild-type (WT) tumors. [44]. While it is accepted that there is resistance to anti-EGFR inhibitors in KRAS mutant patients, data show that patients with KRAS G13D mutation could benefit from EGFR inhibitors [44,45].

## 4. Clinical Factors Associated with RAS Mutation

KRAS mutations are observed in approximately 45% of patients diagnosed with advanced CRC, whereas these mutations are detected in 15–37% of early-stage cases [46,47].

### 4.1. Tumor Sidedness

The relationship between tumor sidedness and the frequency of KRAS mutation is controversial. In the RASCAL 1 and RASCAL 2 studies, which provide extensive information about the relationship between KRAS mutation and clinical factors, no relationship was found between tumor sidedness and KRAS mutation [28,29]. A meta-analysis encompassing 17 studies published in 2019 and another meta-analysis that included approximately 16,000 patients published in 2020 demonstrated that the frequency of KRAS mutations is higher in right-sided CRC [48,49]. Conversely, a subsequent meta-analysis conducted in 2021, which analyzed approximately 6700 patients with metastatic CRC, revealed that the frequency of KRAS mutations was statistically significantly higher in left-sided tumors [50]. The variability in the right and left colon definitions across these studies, along with the inclusion of the rectum in some analyses, further complicates the interpretation of the results.

### 4.2. Gender and Age

The RASCAL 1 and RASCAL 2 studies and a comprehensive study analyzing RAS mutations in 1720 patients identified no correlation between gender and the frequency of RAS mutations. However, a meta-analysis published in 2015 that included five clinical studies revealed that the frequency of RAS mutations was higher in females [28,29,51,52]. No relationship was found between age and RAS mutation in RASCAL studies or many retrospective studies [28,29]. To date, gender has not been shown to have an impact on RAS mutation frequency and therapeutic approaches in patients with early-stage colorectal cancer. Although the incidence of CRC in individuals aged 50 years and older is stabilizing globally, the frequency of young-onset colorectal cancer (YO-CRC), defined as occurring in individuals younger than 50 years, is rapidly increasing [53,54]. Extensive research has been conducted to investigate the frequency of KRAS mutations in YO-CRC cases reported contradictory results. While some studies reported no significant differences in the frequency of RAS mutations between YO-CRC and later-onset CRC (LO-CRC) cases [55,56,57,58,59], others found a lower frequency of mutations in YO-CRC cases (These studies often compare patients under 50 with patients over 50.) [60,61]. Conversely, one study identified a higher frequency of KRAS mutations in YO-CRC cases [62].

### 4.3. Microsatellit Status

The study presented at the ESMO 2023 congress, which evaluated 8460 patients diagnosed with stage III CRC using the ACCENT/IDEA database, provided substantial insights into the importance of RAS mutations [13]. KRAS mutations were detected in 18.1% of microsatellite-instability-high (MSI-H) tumors and 38.6% of microsatellite-stable (MSS) patients. Within the subset of 7492 MSS tumors, patients harboring KRAS-mutant tumors were predominantly female and exhibited tumors originating in proximal locations, compared to those with double wild-type (DWT) tumors. Conversely, among the 968 MSI-H tumors, KRAS mutations were more frequently observed in male patients and were associated with lower-grade histology compared to patients with DWT.

## 5. Effect of RAS Mutation on Early-Stage CRC Prognosis

Alongside all these investigations for a better understanding of KRAS mutation in CRC, the most crucial data for clinical practice is establishing its role in prognosis and exploring predictive value. However, there are inconsistent results regarding the relationship between KRAS mutation and prognosis in early-stage tumors.

The RASCAL I/II studies found that KRAS mutation is associated with poor prognosis in patients with colon cancer [28,29]. The RASCAL studies included patients across various stages, covering both metastatic and early-stage patients, including stage I disease. The first RASCAL study included 2721 patients recruited from 22 groups from 13 countries [28]. KRAS codon 12 or codon 13 mutations were detected in 37.7% of the tumors. In total, 130 (33.9%) of the 384 patients with Dukes’ stage A, 340 (39.8%) of the 855 Dukes’ stage B, 284 (38.3%) of the 742 patients with a Dukes’ stage C and 78 (35.8%) of 265 patients with Dukes’ stage D tumor had a mutation. Multivariate analysis indicated that the presence of KRAS mutation increased the risk of recurrence (*p* < 0.001) and death (*p* = 0.004). KRAS G12V mutation alone was associated with an increased risk of recurrence (*p* = 0.007) and death (*p* = 0.004), suggesting its independent nature as a more aggressive risk factor than other mutations. This study was significant as it provided the first conclusive evidence in common cancers that distinct gene mutations at the same genomic site can impact outcomes differently. This finding highlighted the complexity of genetic influences on cancer progression.

The RASCAL II study was conducted for more comprehensive analysis after the RASCAL I study, encompassing 3439 cases from 35 centers across 19 nations, with an average follow-up period of 55 months [29]. Among the cases, 16% were classified as Dukes’ stage A, 41% as stage B, 31% as stage C, and 12% as stage D. Mutations in the KRAS gene were detected in 35% of the cases analyzed, with 26% occurring in codon 12 and 9% in codon 13. Notably, 8.6% of all mutations involved the substitution of glycine with valine in codon 12 (G12V). Two distinct methodologies were employed to assess the impact of these mutations. Firstly, specific mutations were compared with other mutation types or WT after adjusting for factors such as Dukes’ stage, age, and center of origin. Subsequently, all mutations’ collective effect was compared to WT’s. KRAS mutations exhibited no significant correlation with various clinicopathological factors, including patient age, gender, tumor location, growth pattern, Dukes’ stage, histological subtype, vascular invasion, or lymphocytic response, indicating that utilizing clinicopathologic features alone are not enough to interpret the nature of the tumor and patient outcomes. Multivariate analysis identified advanced Dukes’ stage, patient age, and the G12V mutation as significant predictors of poorer prognosis. Further investigation focused on the impact of the G12V mutation, revealing noteworthy reductions in both disease-free survival (DFS) and overall survival (OS). The influence of this mutation seemed more pronounced in Dukes’ C cancers than Dukes’ B tumors. RASCAL studies did not report data from univariate analysis, and their multivariate analysis did not include tumor site, MSI status, and grade in all patients.

In the CKVO 90–11 study, which compared the effectiveness of fluorouracil/levamisole and fluorouracil/levamisole/leucovorin in patients with stage III colon cancer, no relationship was found between KRAS mutation and DFS [63]. A retrospective study showed that KRAS and BRAF mutations had no negative prognostic effect in stage II and III colon cancer [64]. Similarly, results from CALGB 89803 could not demonstrate the significant impact of KRAS mutation on prognosis either [65]. In the post hoc analysis of the PETACC-8 study, which evaluated the effectiveness of adding cetuximab to adjuvant treatment in patients diagnosed with stage III colon cancer, a significant increase in the risk of relapse and poor DFS was detected in codon 12 mutations and a borderline significant increase in codon 13 mutations [66,67]. The same analysis showed that KRAS mutation had no prognostic significance in MSI tumors. Similarly, in a translational study on PETACC-3, EORTC 40993, and SAKK 60-00 trials of patients with stage II and III resected colon cancer, there was no significant difference in DFS between patients with WT and KRAS mutant tumors, whether unstratified or stratified by stage or MSI status [68]. KRAS mutation status did not predict OS either when analyzed across stages II and III combined or separately, nor within the MSI-Low/Stabil subpopulation. Moreover, assessment of the prognostic and predictive value of MSI status, BRAF, KRAS, NRAS, MET, and PIK3CA mutations from NSABP C-07 and C-08 clinical trial patients failed to show any association between KRAS (including KRAS G12V separately) and OS, recurrence and disease-specific survival (SAR) [69]. In the NCCTG N0147 study, which evaluated the addition of cetuximab to chemotherapy (FOLFIRI) in adjuvant treatment, no effect of the addition of cetuximab was observed as in the PETACC-8 study, while patients with KRAS mutation had a poorer 3-year DFS [70]. Emerging evidence from large phase III trials, such as PETACC8 and N0147, shed light on the potential negative prognostic value of RAS mutations in stage III disease [66,70]. It is important to note that both of these studies included a subset of patients who received cetuximab in addition to adjuvant chemotherapy. Considering the detrimental effect of anti-EGFR agents on RAS mutant tumors, the results from these studies may have shown a more pronounced negative prognostic effect of KRAS.

In the QUASAR study—primarily focused on stage II patients, constituting 91% of the total cohort—patients with KRAS mutations had a notably higher risk of recurrence than those with KRAS WT [71]. In a comprehensive analysis of 1732 cases combining the QUASAR 2 study and Australian CRC samples, KRAS mutations were found to be particularly linked to unfavorable outcomes in MSS tumors. Interestingly, those harboring KRAS mutations in MSI tumors exhibited better prognoses than MSS tumors with KRAS WT or BRAF WT [72]. In a retrospective study evaluating the effectiveness of cetuximab added to FOLFOX treatment in adjuvant therapy in patients diagnosed with stage III colon cancer, it was shown that while KRAS mutation was a poor prognostic marker in MSS tumors, it was not a prognostic marker in MSI tumors [73]. In a prospective study conducted in Japanese patients with stage I–III CRC, KRAS mutation was shown to be associated with poor DFS and OS, independent of MSI status [74]. In another study published in 2015, the presence of type 3 tumor (MSS or MSI-low, non-CIMP, BRAF wt, KRAS mutant), including the presence of KRAS mutation, was evaluated as a negative factor in terms of DFS [75]. A recent pooled analysis of ACCENT/IDEA, representing the largest database to date, investigated the prognostic impact of KRAS exon 2 mutations (codon 12 and 13), BRAFV600E mutations and MSI status in patients with surgically resected stage III CC receiving adjuvant treatment [13]. Of 8460 patients, 968 (11.4%) were MSI-H, and 7492 (88.6%) were MSS. Overall 36.2% of the patients had KRAS exon 2 mutation (26.6% in codon 12 and 8% in codon 13). In the MSS group, KRAS mutations were associated with shorter time to recurrence (TTR) (adjHR:1.31, *p* < 0.0001) and OS (adjHR:1.29, *p* < 0.0001) compared with DWT patients. In contrast, in the MSI-H group, KRAS mutations showed no prognostic effect in TTR and OS. However. KRAS mutations were associated with shorter SAR in both MSI-H (adjHR:1.81, *p* = 0.017) and MSS (adjHR:1.15, *p* = 0.0136) groups of patients. Studies examining the relationship between KRAS mutation and prognosis in patients with early-stage colorectal cancer are shown in Table 1.

Due to the prognostic and predictive role of RAS mutation, molecular analysis is requested as a reflex test in advanced colorectal cancer patients, but unfortunately it is not analyzed in early-stage cases because it has no predictive role and its prognostic role is controversial. One of the possible reasons for this situation is the existence of centers where molecular analysis cannot be performed. If molecular analysis is performed as a standard in all centers, it will both pave the way for clinical studies to be conducted in this field, and it will be possible to conduct research on which mutations are detected more frequently in early-stage cases and targeted treatments for these mutations.

The prognostic role of RAS mutations in early-stage colorectal cancer cases should be investigated with multicenter, prospective studies. However, until these studies are completed, studies that can provide rapid results can be designed by utilizing bioinformatics databases in the light of data obtained over many years.

## 6. Conclusions

The potential prognostic roles of KRAS in the early stages remain areas of active investigation. As KRAS-targeted therapies are being developed and their indications are expanding, there is a need for studies investigating their prognostic role in early-stage patients. Our study is important in that it draws attention to the prognostic importance of KRAS mutation in early-stage colorectal cancer (an area of controversy and lack of data). After the development of KRAS-targeted therapies, it is possible to plan to define its role in the early stage and conduct clinical studies at this stage for the future. The review has some limitations. Our most important limitations are that our study is not a systematic review and that there are few randomized controlled trials on the subject we examined.

## Figures and Tables

**Table 1:** Studies examining the relationship between KRAS mutation and prognosis in patients with early-stage colorectal cancer.

Study	Kras Eligible Patient No. Stage/Location	Treatment	Outcome/Comment
[65] Ogino, 2012 (CALGB 89803)	N = 508 Stage III Colon	5FU/LV vs. IFL	KRAS mutations are not associated with DFS, RFS, or OS. The effect of KRAS mutation on patient survival did not significantly differ according to MSI status. Inclusion of adjuvant irinotecan may have a detrimental effect and diminish the prognostic significance of KRAS mutations.
[66] Blons, 2014, [67] Taieb, 2016 (PETACC-8)	N = 1776 Stage III Colon	FOLFOX ± cetuximab	KRAS is related to shorter DFS and TTR. In subgroup analysis, this prognostic effect of KRAS was restricted to left-sided tumors and codon 12 mutations. KRAS mutation is associated with shorter DFS and OS in MSS but not in MSI patients. The pronounced prognostic effect of KRAS in distal tumors can be explained by the higher prevalence of MSI in this location. The potential detrimental impact of adding adjuvant cetuximab may have amplified the results for KRAS mutant tumors.
[68] Roth, 2010 (PETACC-3, EORTC 40993, SAKK 60-00)	N = 1299 Stage II–III Colon	LV5FU2 vs. FOLFIRI	KRAS mutations are not associated with RFS or OS. Inclusion of adjuvant irinotecan may have a detrimental effect and diminish the prognostic significance of KRAS mutations.
[69] Gavin, 2012 (NSABP C-07/08)	N = 2158 Stage II–III Colon	NSABP C-07: 5FU/LV ± oxaliplatin NSABP C-08: FOLFOX ± Beva	KRAS mutations are not associated with recurrence, SAR, or OS. Included KRAS mutations in codons 59 and 61.
[70] Huang, 2014	N = 146 Stage III Colon	FOLFIRI ± cetuximab	KRAS mutations are associated with poor DFS. The addition of cetuximab showed a trend toward improved DFS and OS regardless of KRAS status.
[71] Hutchins, 2011 (QUASAR)	N = 1583 Stage II–III Colorectal	Observation vs. 5FU/LV	The risk of recurrence is significantly higher for KRAS mutant tumors. KRAS wild and MMR tumors have intermediate prognosis, whereas KRAS mutant tumors exhibit the highest recurrence rate. There is no predictive effect of KRAS on adjuvant ChT benefit. The majority of patients had stage II tumors (91%) and included rectal cancers (28%) Adjuvant regimen is single agent 5FU. Small number of patients in the observation arm. (Possible bias evaluating prognostic effects regardless of adjuvant ChT)
[72] Domingo, 2018 (QUASAR-2 + Australian database)	N = 807 Stage II–III Colorectal	QUASAR-2: Capecitabine ± Beva Australian database Neoadjuvant or adjuvant 5FU-based ChT/CrT	Significant multigene prognostic model including KRAS/BRAF. (It is significant only in stage III tumors). KRAS mutation is significantly associated with poor RFS. KRAS mutation is associated with a poor prognosis in MSS tumors but a better prognosis in MSI tumors. Included rectal cancers. The prognostic impact of KRAS on RFS was more pronounced in distal tumors regardless of MSI status. This can be explained by the higher prevalence of MSI status in proximal location.
[73] Sinicrope 2015 (N0147)	N = 2905 Stage III Colon	FOLFOX ± cetuximab	KRAS mutation is significantly associated with poor DFS, TTR, and OS. KRAS is a prognostic marker in MSS but not in MSI patients. Poor OS for KRAS mutant tumors in distal cancers. In proximal tumors, the impact of KRAS mutations was less pronounced. The potential detrimental impact of adding adjuvant cetuximab may have amplified the results for KRAS mutant tumors.
[74] Kadowaki, 2015 (Saitama cancer center)	N = 812 Stage I–II–III Colorectal	5FU-based ChT and observation	KRAS mutation is associated with poorer DFS and OS. (Independent of MSI status) KRAS mutations were significantly more frequent in females (43%) than in males (35%). KRAS mutations were slightly more common in distal tumors (40%) than in proximal tumors (31%). (Not statistically significant) Included rectal cancers and mutations other than codons 12 and 13. (Exon 2 and 3 mutations were included)
[75] Phipps, 2015 (Seattle registry)	N = 1894 Stage I–IV Colorectal	5FU-based ChT and observation	Type 3 tumors had higher disease-specific mortality. (Type 3 tumor = MSS or MSI-low, non-CIMP, BRAF wt, KRAS mutant) Molecular analyses based on combined subgroups. KRAS mutations were not evaluated separately. Included stage IV patients and rectal cancers.
ChT Beva DFS MSS MSI OS SAR TTR			

## Data Availability

No new data were created or analyzed in this study.
